# Pioneering plant virology research for a healthier future: a summary of the 2025 American Society for Virology (ASV) Plant Virology Satellite Symposium

**DOI:** 10.1128/jvi.01283-25

**Published:** 2026-07-10

**Authors:** Anna E. Whitfield, Olufemi J. Alabi, Juliana Freitas-Astúa, Gesa Hoffman, Marco Incarbone, Fanfang Li, Peter Moffett, Nicole F. Steinmetz

**Affiliations:** 1 Emerging Plant Disease and Global Food Security Cluster, Department of Entomology and Plant Pathology, North Carolina State University 169129, Raleigh, North Carolina, USA; 2 Department of Plant Pathology & Microbiology, Texas A&M AgriLife Research and Extension Center 57804 , Weslaco, Texas, USA; 3 Embrapa Cassava and Fruits, Cruz das Almas, Brazil; 4 Instituto Biológico 245146, São Paulo, Brazil; 5 Max Planck Institute of Molecular Plant Physiology (MPIMP) 28322, Potsdam, Germany; 6 State Key Laboratory for Biology of Plant Diseases and Insect Pests, Institute of Plant Protection, Chinese Academy of Agricultural Sciences 243827, Beijing, China; 7 Centre SÈVE, Département de Biologie, Université de Sherbrooke 98630, Sherbrooke, Québec, Canada; 8 Department of NanoEngineering, University of California, San Diego 8784, La Jolla, California, USA; 9 Center for Nano-ImmunoEngineering, University of California, San Diego 8784, La Jolla, California, USA; 10 Shu and K.C. Chien and Peter Farrell Collaboratory, University of California, San Diego 8784, La Jolla, California, USA; 11 Department of Bioengineering, University of California, San Diego 8784, La Jolla, California, USA; 12 Department of Radiology, University of California, San Diego 204798, La Jolla, California, USA; 13 Institute for Materials Discovery and Design, University of California, San Diego 8784, La Jolla, California, USA; 14 Moores Cancer Center, University of California 8784, La Jolla, California, USA; 15 Center for Engineering in Cancer, Institute of Engineering in Medicine, University of California, San Diego 548962, La Jolla, California, USA; Indiana University Bloomington, Bloomington, Indiana, USA

**Keywords:** plant virus, American Society for Virology (ASV), agricultural viruses, host resistance, emerging diseases, biotechnology

## Abstract

The American Society for Virology (ASV) 2025 Annual Meeting, held from 14 to 17 July at the Palais des congrès de Montréal and hosted by McGill University, featured a dedicated Plant Virology Satellite Symposium entitled “Pioneering Plant Virology Research for a Healthier Future.” This symposium emphasized the inclusion of topics of significance to early-career scientists. The program highlighted advances across diverse areas of plant virology, including mechanisms of transgenerational virus transmission, the economic and biological impacts of grapevine viruses on viticulture, emerging plant virus vectors, and innovative applications of plant viruses as nanomaterials for cancer immunotherapy. Additionally, emerging research on small functional proteins encoded by plant viruses and plant defense mechanisms underscored the complexity of viral genomes and host interactions. The symposium showcased cutting-edge approaches and strategies aimed at addressing global challenges in plant health and food security.

## INTRODUCTION

The American Society for Virology (ASV) has long championed the advancement of virological research and education across diverse biological systems. Its annual meetings serve as a global forum for disseminating cutting-edge discoveries and fostering interdisciplinary collaboration. The 2025 ASV Annual Meeting, held from 14 to 17 July at the Palais des congrès de Montréal and hosted by McGill University, featured a dedicated Plant Virology Satellite Symposium entitled *“*Pioneering Plant Virology Research for a Healthier Future*.*” This symposium was conceived from an interactive plant virology luncheon at the 2024 ASV Meeting in Columbus, Ohio, where participants identified priority research areas critical to the future of plant virology, with an emphasis on ensuring the inclusion of early-career voices in shaping the program. This discussion emphasized emerging diseases, vector biology, host resistance mechanisms, and innovative applications of plant viruses in biotechnology as areas of emphasis for the satellite symposium.

Plant viruses represent a growing threat to global food security, causing billions of dollars in annual losses to major crops such as cereals, fruits, and vegetables. Unlike animals, plants lack an adaptive immune system and rely exclusively on innate defenses, including RNA silencing and receptor-mediated recognition, to combat viral infections. To overcome the barrier of the plant cell wall, plant viruses have evolved specialized strategies, such as encoding movement proteins that facilitate cell-to-cell and systemic transport within the host. Beyond their role as pathogens, plant viruses have emerged as powerful tools in biotechnology, serving as platforms for nanoparticle-based therapeutics, vaccine production, and precision agriculture applications.

Taxonomically, plant viruses are distributed across two major realms: *Riboviria*, encompassing RNA and reverse-transcribing viruses, and *Monodnaviria*, which includes single-stranded DNA viruses. The majority of plant viruses belong to the kingdom *Orthornavirae* and possess positive-sense single-stranded RNA genomes (reviewed in reference 1). While these viruses share the common feature of replicating in plant hosts, they are embedded within the broader virological classification, making them valuable models for understanding virus-host interactions. Historically, plant virology has been at the forefront of major scientific breakthroughs. The first infectious agent to be termed a “virus” was tobacco mosaic virus (TMV), described in 1898, which not only revolutionized virology but also led to foundational discoveries such as RNA viral genomes being infectious and RNA interference as an antiviral defense mechanism. Since then, research on plant viruses has continued to illuminate the fundamental principles of molecular biology, pathogenesis, immunity, and evolution.

The 2025 Plant Virology Satellite Symposium reflected the rich legacy of groundbreaking research with plant viruses while addressing contemporary challenges and opportunities in the field. Talks from leading experts on topics ranged from transgenerational virus transmission and grapevine virus epidemiology to virus-based nanotechnologies and the discovery of small functional viral proteins (Table 1). Collectively, these presentations underscored the dynamic nature of plant virology research and its relevance to global agriculture, biotechnology, and One Health. This review synthesizes the key themes discussed during the symposium and explores their implications for advancing plant virology toward a healthier and more sustainable future. The full ASV meeting program also featured plenary and state-of-the-art presentations on plant viruses by Sir David Baulcombe and Tessa Burch-Smith.

**TABLE 1:** List of speakers and presentation titles at the 2025 ASV Plant Virology Satellite Symposium “Pioneering Plant Virology Research for a Healthier Future”

Speaker	Presentation title
Gesa Hoffmann	Mapping transgenerational barriers in plant virus transmission
Nicole F. Steinmetz	Plant virus-based therapeutics for human and plant health
Fanfang Li	Plant viruses encode additional functional proteins
Juliana Freitas-Astúa	Transmission biology of emerging mite-borne viruses
Olufemi J. Alabi	Grapevine-infecting viruses—effects on the plant hosts and wine
Peter Moffett	Resistance and antiviral defense mechanisms in plants

## MAPPING TRANSGENERATIONAL BARRIERS IN PLANT VIRUS TRANSMISSION

Viruses are ubiquitous genetic parasites that inhabit and influence every biome on Earth. One hallmark of an adapted virus is its ability to exploit the host machinery for replication and to transmit between hosts. Plant viruses use essentially two non-mutually exclusive routes of transmission. On one hand, horizontal transmission occurs through vectors like insects, mites, and nematodes from infected donor plants to susceptible recipient plants in proximity (2). On the other hand, vertical transmission from the parent to progeny through seeds enables the spread of viral infections (i) over time from one generation to the next, synchronizing virus disease with its host growth cycle, and (ii) over great geographic distances by pollen and seed dispersal or global seed trade. Of note, here we refer to vertical transmission through sexual reproduction and not through vegetative propagation.

A few hundred viruses are known to be transmitted through seeds (3–5), albeit typically at low frequencies. A systemic assessment of the vertical transmission capability of most plant viruses is lacking and is a daunting task as transmission rates can depend on the virus strain, host species, infection timing, and environmental factors. Although the term vertical transmission is often used synonymously with seed transmission, both terms are only loosely defined and do not specify whether viral transgenerational infection occurs (i) in gametes before fertilization, (ii) in the developing embryo after fertilization, or (iii) in the germinating seedling through the infected seed coat. It is important to note that gamete-mediated transmission imposes a severe bottleneck on a virus, as a single founder cell, and its viral complement give rise to all ensuing populations (6, 7). Thus, understanding the tissue-specific tropism of plant viruses in relation to reproductive structures, as well as the extent of invasion into male and female gametes, offers insights into infection strategies, cell-type-specific antiviral immunity, and targeted control measures that reduce transmission without driving resistance-breaking.

The earliest potential entry point for a plant virus into the germline-track precursor cells is the stem cells within the shoot apical meristem (SAM). Most pathogenic plant viruses, however, are effectively excluded from SAMs and from the subsequently developed germline, indicating that many host plants are able to suppress vertical transmission partially or completely (8). It is assumed that virus SAM exclusion and lack of vertical transmission are directly connected, but this remains to be causally and temporally established, ideally through technically challenging live microscopy and developmentally staged infection experiments. Furthermore, these approaches could reveal where and when viruses enter—or are excluded from—the female and male germlines, a complex phenomenon which is likely highly dependent on host and virus species. The widespread and conserved ability of plants to restrict virus vertical transmission suggests the presence of potent transgenerational antiviral barriers that are established in plant SAM stem cells and act throughout flower development and sexual reproduction, allowing even severely infected plants to produce healthy progeny. For several virus-host combinations, somatic RNAi factors, hormones, transcriptional regulators, and in turn the viral suppressors of RNAi silencing were found to influence SAM invasion and vertical transmission rates (3, 9–14). Yet, despite their importance for host-virus interactions and crop protection, these transgenerational barriers remain very poorly understood at the spatial, temporal, and mechanistic levels. A major future challenge will be to determine which plant genes and which corresponding molecular mechanisms regulate germline antiviral defense. Determining where and when during plant sexual reproduction these are active will provide important information to design strategies to prevent virus vertical transmission in agriculture. Interestingly, the broad effectiveness of these antiviral barriers suggests that they are largely immune to viral suppression, as recently exemplified by the RNAi-dependent exclusion from stem cells of viruses that effectively suppress RNAi in the rest of the plant (11). These “super-defenses” guarding arguably the most consequential cells in the plant are of obvious fundamental and applied interest. For example, recent research suggests a key role for stem cell- and germline-specific RNAi factor AGO5 in limiting vertical transmission in gametophytes and gametes, providing a compelling first report of germline antiviral defenses (15).

An equally important part in the vertical transmission process is played by the virus itself: how it interacts with the host and its loosely termed “lifestyle.” The little data available suggest that viruses causing less pathogenic and more latent/persistent infections tend to be more efficiently vertically transmitted (8), as has also been shown in accelerated viral evolution experiments (16). As mentioned above, this is highly dependent on the virus strain, suggesting a pronounced role for viral genetics. It may be that, from the virus’s point of view, the ability to persist in plant lineages through generations comes at the cost of the speed and severity of infection, which in turn may reduce the efficiency of horizontal transmission through vectors. Moreover, vertical transmission requires the virus to remain infectious in embryos during seed dormancy and, in perennial plants, to access the germline year after year. Assessing the rates and patterns of virus vertical transmission in wild plants could provide exciting new information on virus dynamics in nature. It can be argued that the short-term cycles of intensive agriculture may favor severe, horizontally transmitting viruses, while wild populations and ecosystems may present more sustainable, long-term viral communities that rely heavily on vertical transmission.

So far, mechanistically addressing the enigma of vertical transmission has been hampered by the small number of cells involved and the scarcity of suitable experimental systems that are amenable to large-scale genetic screens and detailed mechanistic studies. Hopefully, the coming years will see momentous advances in our understanding of how viruses interact with plant germlines and how they are allowed or not to pass from one generation of their host to the next.

## PLANT VIRUS-BASED THERAPEUTICS FOR HUMAN AND PLANT HEALTH

Repurposing plant viruses as nanomaterials for applications in both human and plant health represents a pioneering and novel approach to plant virology. With a toolkit of chemical biology methods, strategies to functionalize plant viruses with active cargos, including therapeutics, pesticides, and epitopes, have been developed (17). Structure–function studies utilizing a defined set of plant viruses with distinct material properties (size, shape, and functionality) led to unique discoveries. On one front, plant viruses can serve as potent cancer immunotherapies; on the other, they have been developed as plant virus-based nanotechnologies for crop protection from nematodes.

Cowpea mosaic virus (CPMV) is a non-enveloped 30 nm-sized icosahedron with a bipartite positive-sense ssRNA genome (18), and CPMV acts as a highly potent intratumoral immunotherapy. In intratumoral immunotherapy, an immunomodulatory agent—here CPMV—is administered directly into a tumor or metastatic site to stimulate the immune system and reverse immunosuppression induced by aggressive tumors. CPMV—but not other plant viruses—was shown to be highly potent in this application (19, 20). Robust efficacy has been demonstrated across murine tumor models, including melanoma, ovarian, breast, glioma, and colon cancers in various anatomical locations (subcutaneous, dermal, intraperitoneal, intracranial, and pulmonary metastases). Results were consistent across mouse strains, sexes, and ages. Importantly, CPMV induces durable antitumor immunity, with rechallenge experiments confirming long-term immunological memory (21). Mechanistically, CPMV modulates the tumor microenvironment (TME) by activating innate immune populations—M1 macrophages, N1 neutrophils, dendritic cells, and NK cells—leading to tumor cell killing and efficient antigen presentation. This primes adaptive immunity mediated by tumor antigen-specific CD8+ and CD4+ T cells (22).

Translational relevance has been validated in companion canine cancer patients. In one trial, intratumoral CPMV administered in the neoadjuvant setting (two injections prior to surgery) induced regression in both injected and non-injected lesions with impressive survival data; the efficacy was also correlated with the mechanism of action, and histology confirmed immune infiltration of neutrophils and T and B lymphocytes (23). Taken together, these findings position CPMV as a first-in-class plant virus-based immunotherapy poised for clinical translation.

**Fig 1:**
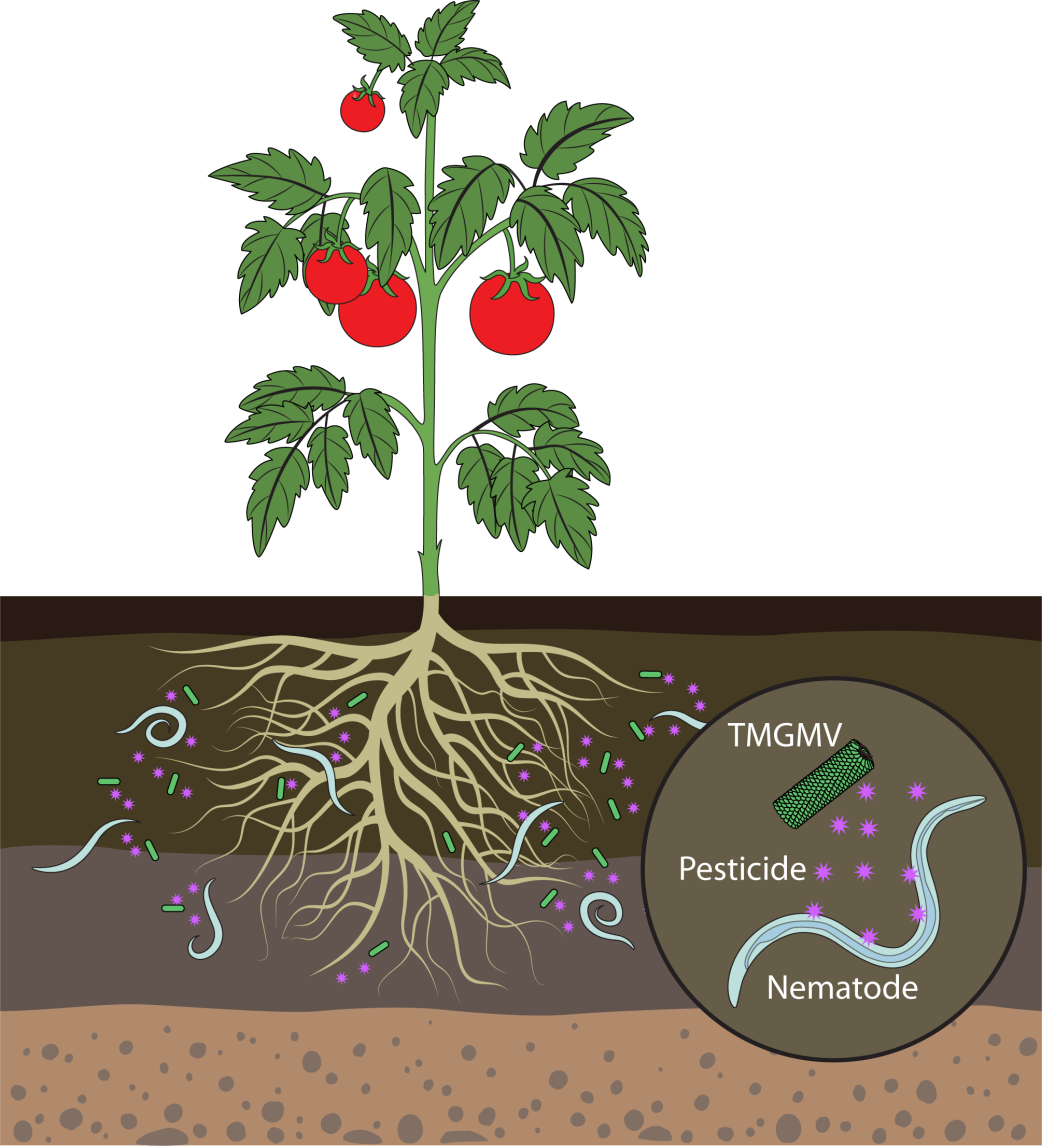
Cartoon showing tobacco mild green mosaic virus (TMGMV, green) delivering pesticides (purple) to nematodes in soil.

In a different application, tobacco mild green mosaic virus (TMGMV), a rod-shaped plant virus (300 × 18 nm), has been developed as a nanocarrier for delivering nematicides to control plant-parasitic nematodes Fig. 1. Early studies showed that TMGMV can encapsulate small molecules such as crystal violet via electrostatic loading into its negatively charged interior channel (measuring 4 nm in width); the cargo-laden TMGMV nanoparticles demonstrated efficacy against *Caenorhabditis elegans* in liquid culture (24). Importantly, plant parasitic nematodes feed on the roots of crops—thus, to tackle the problems at its roots, formulations of nematicides must have good soil mobility. This, in fact, is a major barrier to success—farmers have to apply a high dose of current nematicides to ensure killing of the pests; however, this overdosing and not-targeted approach results in 90% of the pesticides being lost and harming the environment. Interestingly, TMGMV exhibits excellent soil mobility profiles compared to other plant viruses and synthetic nanomaterials, reaching depths of up to 30 cm and enabling effective rhizosphere delivery (25). Given the success of rhizosphere targeting of TMGMV, chemical strategies for pesticide loading were developed (26). To advance translation in agriculture, scalable and one-pot synthesis methods are needed, and toward that goal, the thermal reshaping of the TMGMV soft matter rods or even just their coat proteins into solid nanoparticles (SNPs) spanning nano-to-micrometer scale can be utilized: small-molecule cargo (such as pesticides) can be packaged during the thermal shape transition (27). It is truly intriguing that nature rod-shaped nanoparticles can be morphed into spherical materials through heat annealing.

Together, these examples highlight the potential impact that plant virus nanotechnology could have on human, veterinary, and plant health. Viruses are the most abundant biological entities on the planet, with an estimated 10^31^ viruses on Earth. It is clear that there is still much to learn about the viral diversity to protect the population from infection. This biodiversity, however, also offers tools for biotechnology. The Plant Virus Database (PVD) lists >3,000 virus species, but only a handful of plant viruses are being studied in the context of biotechnology—the above examples highlight that distinct plant viruses have unique properties—to fully capitalize on plant viruses as next-generation platform technology; thus, we need to begin systemic structure-function studies.

## PLANT VIRUSES ENCODE ADDITIONAL FUNCTIONAL SMALL PROTEINS

**Fig 2:**
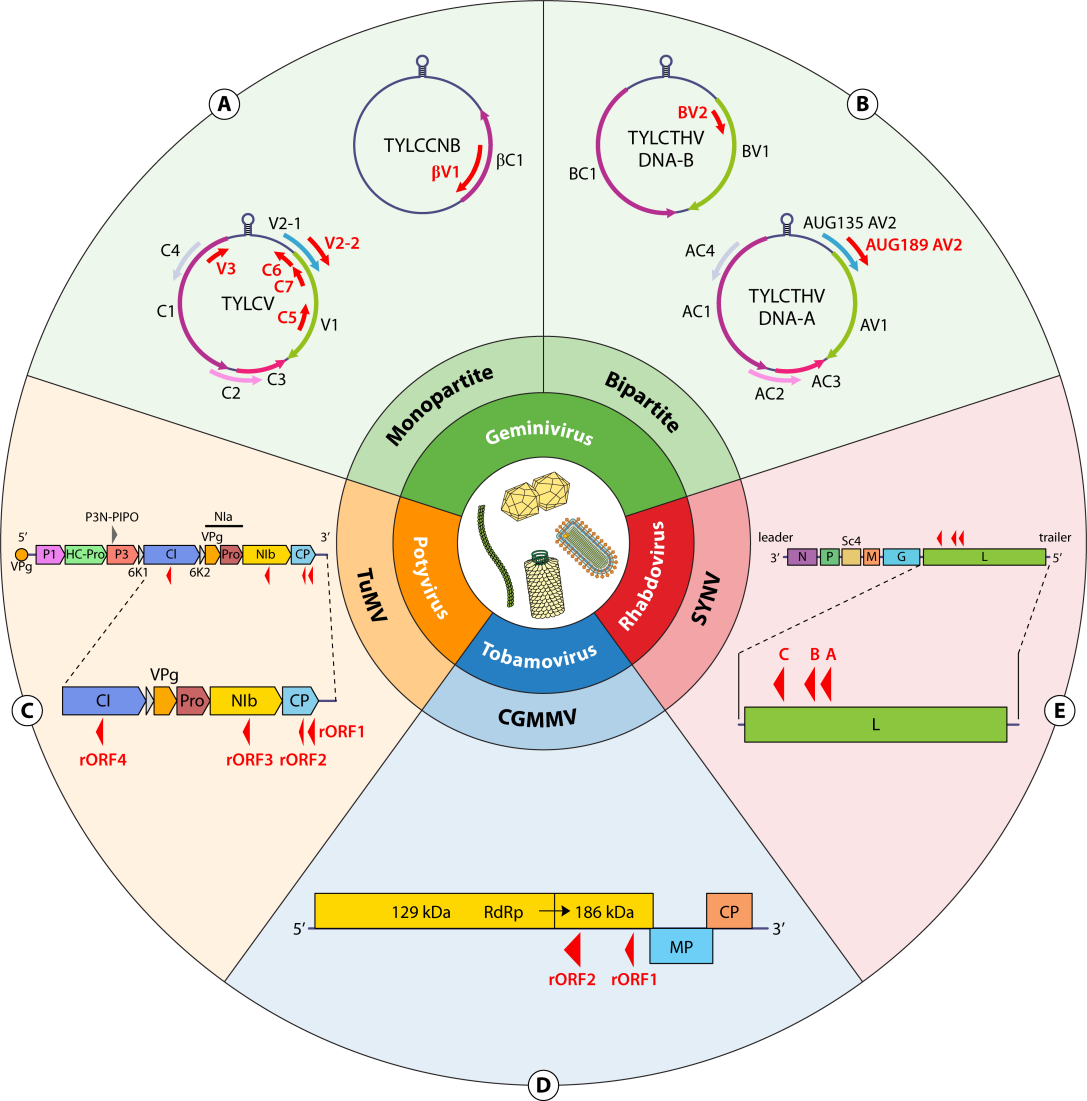
Recently identified small open reading frames (ORFs) encoded by various plant viruses. (**A**) Genome organization of tomato yellow leaf curl virus (TYLCV, a monopartite geminivirus) and an associated betasatellite. TYLCV encodes six known ORFs (C1, C2, C3, C4, V1, and V2-1), with five additional ORFs recently described (V3, C5, C6, C7, and V2-2). Tomato yellow leaf curl China betasatellite (TYLCCNB) encodes the typical βC1 protein and a newly identified βV1 protein. (**B**) Genome organization of tomato yellow leaf curl Thailand virus (TYLCTHV, a bipartite geminivirus). The DNA-A component of TYLCTHV contains six known ORFs (AC1, AC2, AC3, AC4, AV1, and AV2-1/AUG135), along with the newly identified AV2-2/AUG189. The DNA-B component encodes two known ORFs (BV1 and BC1) and one newly identified ORF (BV2). (**C**) Genome organization of turnip mosaic virus (TuMV, a potyvirus). TuMV encodes 11 known proteins (P1, HC-Pro, P3, P3N-PIPO, 6K1, CI, 6K2, VPg, NIa, NIb, and CP). Additionally, rORF1 to rORF4 are derived from its negative-stranded RNA. (**D**) Genome organization of cucumber green mottle mosaic virus (CGMMV, a tobamovirus). CGMMV encodes four known proteins (129 kDa RdRp, 186 kDa RdRp, MP, and CP), with rORF1 and rORF2 translated from its negative-stranded RNA. (**E**) Genome organization of Sonchus yellow net virus (SYNV, a rhabdovirid). SYNV contains six known ORFs on the positive-stranded antigenomic RNA (+agRNA), encoding the N, P, Sc4, G, M, and L proteins. Putative small ORFs (A, B, and C) are present on the genomic RNA (gRNA, −RNA). All recently identified ORFs are highlighted with red arrows.

Plant viruses have long been known for their compact genomes and limited coding capacity. Traditional virology focused on canonical open reading frames (ORFs) encoding proteins larger than 10 kDa, such as viral-encoded RNA-dependent RNA polymerases (RdRps), movement proteins (MPs), and coat proteins (CPs). However, recent advances in genomics, proteomics, and bioinformatics have revealed that plant viruses using non-canonical translational strategies encode numerous small, previously overlooked proteins that play critical roles in viral infection and host interactions Fig. 2 (28, 29).

Geminiviruses (family *Geminiviridae*) are among the largest and most economically important groups of plant-infecting DNA viruses, with over 540 recognized species (30). Although these ssDNA viruses were traditionally thought to encode only four to eight proteins, recent studies have revealed multiple small ORFs that encode functional proteins. For instance, tomato yellow leaf curl virus (TYLCV) encodes V3, C5, and C7, which are involved in viral movement, silencing suppression, and pathogenesis, respectively (31–36) (Fig. 2). C5 proteins from several geminiviruses localize to plasmodesmata and facilitate intercellular movement by interacting with the movement protein V2 (36). Tomato leaf curl China virus and TYLCV encode a novel C6 protein with an internal mitochondrial targeting signal, which is localized in the mitochondria (37). Additionally, the βV1 protein, encoded by tomato yellow leaf curl betasatellite, localizes to the endoplasmic reticulum (ER) and induces ER aggregation and the unfolded protein response (38, 39). Although βV1-mediated induction of bZIP17/28 is critical for viral infection, ER stress-induced autophagy can target βV1 for degradation through a negative feedback loop, thereby attenuating viral infection (39). Notably, Chiu et al. used tomato yellow leaf curl Thailand virus (TYLCTHV) as a model and identified previously unannotated functional ORFs (40). The conserved downstream AUG sites in AV2 and BV2 produce distinct AV2 protein isoforms and an ER/plasmodesmata-localized BV2 protein, respectively. Mutational analyses confirmed that both AV2 isoforms and BV2 are essential for full pathogenicity in tomato, demonstrating the critical role of these hidden viral ORFs in mediating viral infectivity by generating distinct protein/isoforms via viral alternative AUG start sites (40). Similarly, TYLCV V2 contains two initiation codons (ATG1/V2-1 and ATG2/V2-2), yielding two distinct protein isoforms that collaboratively enhance virulence and host manipulation (41) (Fig. 2). Together, these findings highlight how geminiviruses maximize their genome coding capacity through overlapping ORFs and diverse translation initiation sites to synthesize a broader repertoire of functional proteins.

Potyvirids (family *Potyviridae*), the largest family of plant positive-sense RNA viruses, typically express their genomes through a polyprotein that is proteolytically processed into functional units. However, recent findings have revealed that potyvirids also encode additional functional small proteins derived from their negative-stranded RNA (−RNA) (Fig. 2), challenging the conventional view that −RNA lacks coding potential (42, 43). Gong et al. identified multiple conserved small reverse ORFs (rORFs) in the −RNA of both plant and animal positive-sense RNA viruses (43). In turnip mosaic virus (TuMV), rORF2—detected via ribosome profiling and mass spectrometry—interacts with the viral RdRp within replication complexes and is essential for viral replication in *Arabidopsis* and *Nicotiana benthamiana* (43). Beyond potyvirids, cucumber green mottle mosaic virus (CGMMV) and tobacco mosaic virus (TMV) (genus *Tobamovirus*, family *Virgaviridae*) encode rORF1 and rORF2 on the viral −RNA (Fig. 2). CGMMV and TMV rORF2 localize at the cell membrane and in the nucleolus, while rORF1 colocalizes with peroxisomes. Of note, CGMMV rORF1 was found to interact with PEX3.1, a conserved multifunctional peroxisomal transmembrane protein that is involved in the insertion of peroxisomal membrane proteins, which guides rORF1 to target to peroxisomes and recruits RdRp to enhance viral replication (44). These results together uncover the existence of additional, previously overlooked proteins encoded in the −RNA strand of positive-sense RNA viruses.

Viruses employ diverse non-canonical translation strategies—such as internal ribosome entry (IRES), leaky scanning, non-AUG initiation, alternative AUG start sites, ribosome shunting, reinitiation, ribosomal frameshift, and stop-codon readthrough—to maximize the coding efficiency of their compact genomes (45). For instance, Chiu et al. revealed that AV2 protein isoforms and BV2 are produced via alternative AUG start sites (41); Gong et al. further demonstrated that SARS-CoV-2, TuMV, and CGMMV utilize IRES elements to hijack host ribosomes and enable translation of these rORF-encoded small proteins, thereby expanding the viral proteome (43, 44).

In addition, a recent study also expanded our understanding of the coding potential of –ssRNA viruses, revealing that their genomic RNA (gRNA) can encode functional small proteins. Using the model plant rhabdovirus Sonchus yellow net virus (SYNV, family *Rhabdoviridae*), Gao et al. first predicted all the ORFs in gRNA and antigenomic RNA (agRNA) and identified three conserved small ORFs (sORFs)—designated A, B, and C—located antisense to the viral L gene in the genome RNA (46). These sORFs encode proteins that localize to the nucleus and cytoplasm and exhibit time-dependent accumulation. Functional characterization demonstrated that these small proteins play critical roles in viral pathogenicity. Specifically, the mutation of the start codon of sORF B delayed SYNV infection, reduced symptom severity, and decreased viral RNA accumulation. Heterologous expression of A and B in a potato virus X (PVX) system enhanced PVX accumulation and symptom severity, indicating their proviral functions. Protein interaction assays revealed that A interacts with SYNV M and P proteins, while B interacts with M, suggesting involvement in viral replication, assembly, or movement. This study challenges the conventional view that –ssRNA viral gRNA serves solely as a template for transcription, highlighting a previously overlooked layer of genomic complexity.

In conclusion, these findings underscore the importance of revisiting viral genomes for hidden coding sequences, and plant viruses encode a diverse array of small functional proteins that play pivotal roles in their life cycle and pathogenicity. The study of these proteins represents a new frontier in plant virology, with profound implications for both basic science and crop protection. However, research on virus-encoded small ORFs faces several challenges: their short length (typically encoding peptides under 10 kDa), low detection efficiency due to limitations of antibody- and MS-based methods caused by low abundance and difficult extraction, frequent overlap with known coding sequences that complicates annotation and functional analysis, and diverse biological roles that are still poorly understood and lack standardized functional assays. These difficulties explain why such small ORFs remained undetected until recent advances in sensitive genomic and proteomic technologies (Fig. 2). Thus, the current understanding likely represents only the tip of the iceberg—future studies are crucial to fully elucidate the functional significance of these hidden viral elements.

## TRANSMISSION BIOLOGY OF EMERGING MITE-BORNE VIRUSES

Plant viruses are transmitted primarily by insects, particularly members of the order Hemiptera. However, the diversity of plant virus vectors extends well beyond insects and includes mites, nematodes, chytrid fungi, and protists. Among these, mites have long been recognized as vectors, although our understanding of their interactions with viruses remains limited. The increasing number of described mite-borne viruses, coupled with the significant economic impact of some of the diseases they cause, highlights the need for renewed attention to this group of vectors. Two main families of phytophagous mites, Eriophyidae and Tenuipalpidae*,* are associated with virus transmission. Members of 10 eriophyid genera transmit viruses belonging to at least eight genera, as diverse as *Tritimovirus* (family *Potyviridae*) and *Emaravirus* (family *Fimoviridae*), to hosts ranging from cereals to stone fruits (47, 48). In contrast, confirmed tenuipalpid vectors are restricted to *Brevipalpus* spp., which transmit viruses of the families *Rhabdoviridae* (genus *Dichorhavirus*) and *Kitaviridae* (genera *Cilevirus* and *Higrevirus*) to a wide variety of hosts (49, 50) and are likely one of the most relevant components directing the convergent evolution of cileviruses and dichorhaviruses (51). Family *Rhabdoviridae* is one of the largest families of viruses, comprising 62 genera and 580 species that infect animals and plants. The only rhabdovirids known to be transmitted by mites are the members of the genus *Dichorhavirus*. By contrast, family *Kitaviridae* is a small virus family, comprising only three genera (*Cilevirus, Higrevirus*, and *Blunervirus*) and 13 species of plant viruses (50). Two major questions associated with the transmission biology of mite-borne viruses were addressed in this symposium. The first concerns the identity of blunervirus vectors, which, until recently, were the only genus within *Kitaviridae* without confirmed vectors. Previous reports suggested both eriophyids and tenuipalpids as potential candidates. Blueberry necrotic ring blotch virus (BNRBV, *Blunervirus vaccinii*) occurs in regions where both groups of mites are present, while tomato fruit blotch virus (ToFBV, *Blunervirus solani*) has been epidemiologically associated with the eriophyid tomato russet mite in Europe and Brazil. Nevertheless, all other kitavirids with characterized vectors were known to be transmitted exclusively by *Brevipalpus* spp. This uncertainty was recently resolved as the eriophyids *Calacarus corymbosi* and *Aculops lycopersici* were shown to transmit, respectively, BNRBV and ToFBV (52, 53). These findings represent a significant advance in understanding blunervirus ecology, especially in light of several new tentative species identified worldwide through high-throughput sequencing (54). Establishing vector identity is essential for elucidating disease epidemiology and developing rational strategies to mitigate emerging pathogens.

The second research question addressed the interaction between *Brevipalpus yothersi* and citrus leprosis virus C (CiLV-C, *Cilevirus leprosis*). Earlier studies suggested a persistent-circulative type of transmission, based on short acquisition (~4 h) and inoculation (~2 h) access periods, transmission competence across all active life stages of the vector (larvae, nymphs, and adults), and a brief latent period of ~7 h (55). In contrast, Clerodendrum chlorotic spot virus (ClCSV, *Dichorhavirus clerodendrii*), also transmitted by *B. yothersi*, exhibits a persistent-propagative strategy, characterized by a longer latent period (~8 days) and transmission restricted to nymphs and adults. Furthermore, while ClCSV induces electron-lucent viroplasms within the nuclei of midgut epithelial and anterior prosomal gland cells, as well as spoke-wheel inclusions in the cytoplasm of *B. yothersi* mites (56), CiLV-C particles are confined to intercellular spaces, indicating a paracellular route of movement within the vector (57). Altogether, these observations initially suggested that dichorhaviruses such as ClCSV replicate within their mite vectors, whereas cileviruses like CiLV-C appeared to circulate without replication. Supporting the propagative nature of mite-dichorhavirus interactions, orchid fleck virus (OFV, *Dichorhavirus orchidaceae*), transmitted by *B. californicus*, has been shown to replicate in its vector (58). In contrast, reports of CiLV-C and citrus leprosis virus C2 (CiLV-C2, *Cilevirus colombiaense*) replication in mites, based on the detection of anti-genomic RNAs (agRNAs) (59), introduced a paradox. To address this, experiments with rigorous biological and technical controls were undertaken. These included comparisons between vector and non-vector mites feeding on CiLV-C-infected plants, fasting before transferring them to non-host plants (*Citrus latifolia*) to exclude the amplification of plant-derived viral agRNAs from gut content, and the use of biotinylated or tagged primers to prevent self-priming artifacts (60). The results revealed that agRNAs were consistently detected only in vector mites, albeit at low but stable levels over 21 days. Furthermore, the sub-genomic-to-genomic RNA ratio in mites was noticeably lower than that observed in plants (61), but consistent with replication. Collectively, these data suggest that CiLV-C replicates in mites, though inefficiently and likely restricted to specialized cells yet to be identified.

Accurate identification of mite vectors and characterization of their interactions with viruses are critical steps in understanding disease ecology and in designing sustainable control strategies. For blunerviruses, recognizing eriophyids as vectors provides a foundation for epidemiological studies and risk assessments of emerging species. For cileviruses and dichorhaviruses, unraveling differences in transmission not only deepens our understanding of virus-vector biology but also provides crucial insights for disease management in economically important crops such as citrus.

## GRAPEVINE-INFECTING VIRUSES: EFFECTS ON THE PLANT HOSTS AND WINE

The global virome of grapevine (*Vitis* spp.) consists of more than 100 viruses in 44 genera and 21 families (62), making it the host with the most number of viruses infecting a single crop. Among them, the causal or associated viruses of grapevine leafroll disease (GLD), fanleaf degeneration and decline, and rugose wood disease complex (63, 64) are globally distributed and considered economically consequential. Grapevine red blotch disease (GRBD) is another important malady of grapevine, especially in the context of North America, due to its widespread nature and epidemics across several states in the United States, Canada, and Mexico (65). The other grapevine viruses have limited distribution and are thus considered of local importance to the regions where they were documented (66).

In the United States, GLD and GRBD are the top two virus disease threats to vineyard prosperity due to their widespread nature, recalcitrance to management, and documented negative impacts on vine health and productivity (67–70). The viruses involved in GLD are collectively called grapevine leafroll-associated viruses (GLRaVs), and six of them have been identified to date, among which GLRaV-3 is the most prevalent. GLRaVs, so-called because Koch’s postulate is yet to be conclusively proven for them *stricto sensu*, belong to three genera in the family *Closteroviridae* (71). Four of the GLRaVs (GLRaV-1, GLRaV-2, GLRaV-3, and GLRaV-4) are assigned to the genus *Ampelovirus*, a taxon of viruses vectored by multiple species of mealybugs and scale insects. One virus each belongs to the genera *Closterovirus* (GLRaV-2) and *Velarivirus* (GLRaV-7). Unlike GLD, GRBD has one definitive causal agent, grapevine red blotch virus (GRBV), an ssDNA virus and prototype of the genus *Grablovirus* (family *Geminiviridae*). The only proven vector of GRBV is the three-cornered alfalfa treehopper (TCAH; *Spissistilus festinus* Say), but observations of GRBV secondary spread in Oregon vineyards devoid of TCAH suggest that additional membracid vectors may be involved (72).

One of the most obvious effects of GLRaVs and GRBV is the foliar reddish discoloration induced in red- or black-fruited wine grape cultivars beginning at the onset of berry ripening (véraison) until berry maturity and leaf fall. Whereas a trained eye may be able to distinguish between foliar reddening due to GLD versus GRBV, the symptom differences may be subtle and can be influenced by vagaries of vine mixed infection status, grapevine cultivar differences, and vine nutritional disorders, among other confounding factors. Interestingly, although equally susceptible to infections by GLRaVs and GRBV, white-fruited wine grape cultivars and several other cultivated *Vitis* species (e.g., *V. labruscana* “Concord”) seldom show obvious foliar discoloration (73), rendering symptoms-based diagnosis impractical. Also, because of the cyclic nature of GLD and GRBD symptoms, wherein diseased vines remain chronically infected although they show symptoms only beginning from véraison to leaf fall, infections may linger before disease is noticeable in the vineyard, thus compounding disease epidemics.

Both GLD and GRBD affect grapevine physiological parameters such as foliar photosynthesis, carbohydrate metabolism, and assimilate translocation (74–81), with these perturbations occurring in concurrence with foliar disease symptoms expression. Multiple studies have also documented the negative impacts of GLRaV-3 and GRBV on the source/sink balance of red- or black-fruited grapevine cultivars, including interference with the expression of genes involved in the biosynthesis of anthocyanin and sugar metabolism (76, 82–84). These changes result in altered berry chemistry parameters such as reduced total soluble solids and anthocyanin accumulation in berries (67, 85–87), which may result in negative impacts on wine quality (67, 83, 87–90) and their sensory attributes (67, 88, 89). The negative effects of GLD and GRBD on vine health and productivity translate into significantly diminished vineyard profitability depending on factors such as vineyard location, initial infection incidence, cultivar, and price penalty for low-quality fruit (70, 91).

So serious is the dual threat of GLD and GRBD that, at the behest of the California Department of Food and Agriculture, the National Academies of Sciences, Engineering, and Medicine (NASEM) recently assembled a group of experts to review the current state of knowledge on both diseases, identify knowledge gaps, and develop recommendations to guide research on the viruses, vectors, and plant hosts, toward advancing strategies for disease management. The NASEM committee’s report (92) identified several research topics and actions that may yield promising management solutions. Among them are the implementation of higher sanitary standards in registered mother blocks using robust and reliable diagnostic methods, development of optimal rouging and replanting schemes and facilitating their implementation, optimizing systemic insecticide usage for better mealybug and TCAH control and investigations into other control methods, implementation of areawide GLD and GRBD vector management programs, and incentivizing growers to participate in these programs. The committee’s report also identified the need to develop models to guide insect vector and disease management on an areawide basis, engage early and mid-career scientists to address specific knowledge gaps on GLD and GRBD management, fund support for longer-term and replicated studies on the identified GLD and GRBD knowledge gaps, form working groups of researchers, industry, and government to facilitate greater sharing and integration of research findings, and implement effective educational and outreach strategies to communicate GLD and GRBD management advances to growers to help boost the adoption of new practices.

## RESISTANCE AND ANTIVIRAL DEFENSE MECHANISMS IN PLANTS

One of the most effective methods for countering virus infections is through genetically encoded host resistance. This includes multiple mechanisms, including a lack of compatibility between virus and host translation factors (93), as well as mechanisms involving active recognition of viral components (94). The latter includes the recognition of viral proteins by plant Nucleotide-binding Leucine-Rich repeat (NLR) proteins. NLR proteins recognize pathogens, including viruses, resulting in a signaling cascade that culminates in pathogen resistance (95). Although many advances have been made in understanding recognition by, and activation of, NLR proteins, less is known about the execution of disease resistance. However, it has been reported that NLR proteins induce a response that targets viral RNAs for translational repression (96, 97), as well as abscisic acid-mediated antiviral responses (98).

Plants also employ antiviral mechanisms based on the recognition of double-stranded RNA (dsRNA), which is generated during the replication of RNA viruses. The best characterized such mechanism is RNA silencing, wherein Dicer-like (DCL) proteins recognize dsRNA and cleave it into virus-derived small RNAs (vsiRNAs). These vsiRNAs are in turn bound to Argonaute (AGO) proteins, which use vsiRNAs to target any homologous single-stranded RNA for endoribonucleolytic cleavage and/or translational repression (99, 100). However, in most experimental models, this mechanism is not effective because plant viruses encode viral suppressors of RNA silencing (VSRs). This presents challenges to studying RNA silencing. This can be overcome by various approaches, including using VSR-defective viruses or by demonstrating increased susceptibility to viruses in plants lacking RNA silencing components (99, 101). An alternative approach involves studying viruses that do not normally infect a particular host. For example, *Arabidopsis thaliana* Col-0 is not infected by potato virus X (PVX), but mutants lacking the *AGO2* and *AGO5* genes are highly susceptible to PVX, demonstrating that RNA silencing can in fact be an effective defense against a wild-type virus (102). This approach has also shown that the *AGO2* gene is highly polymorphic, showing important sequence variation both within and between species, which translates to dramatic differences in the antiviral activity (103). This study suggested that AGO2 and possibly other RNA silencing components may determine the host range in plant-virus interactions and that natural variation in these components may be useful for generating virus-resistant plants.

It is notable that NLRs were discovered relatively early because the genes that encode them are variable between plant cultivars, which led to their eventual cloning. However, such variation in RNA silencing components was discovered only much later and to date, only in one plant species (103). As such, additional antiviral mechanisms may exist in plants that remain poorly characterized. This may be because they are highly effective, resulting in non-host resistance, but leaving no observable plant-virus interaction to study. Alternatively, such mechanisms might be overcome by viruses in their host plants in compatible interactions, and thus, in the absence of natural variation, their existence is not observed. Progress has been made in investigating plant antiviral mechanisms that are analogous to phenomena known to exist in other systems. For example, dsRNA is recognized as a pathogen-associated molecular pattern (PAMP) by different types of proteins in animal systems, including Toll-like receptors (TLRs), NLRs, RIG-I receptors, and protein kinase RNA-activated (PKR) (104). Interestingly, it has been reported that plants also respond to extracellularly applied dsRNA in a manner similar to other PAMP-triggered immunity (PTI) responses (98, 105). Although it is still unknown what type of receptor recognizes dsRNA in plants and whether this occurs inside or outside the plant cell, it has been shown that plants defective in the BAK1 protein are more susceptible to virus infection (106, 107). The BAK1 protein acts as a partner for many transmembrane pattern recognition proteins (PRR) that detect PAMPs, suggesting that BAK1 may interact with an unknown PRR that recognizes viral dsRNA. If and how such RNA might end up outside the cell, however, has yet to be determined. At the same time, it has been proposed that such RNA might be detected intracellularly (108). Intracellular perception of dsRNA in animal cells results in global repression of protein translation through phosphorylation of translation factors (104). Interestingly, DNA virus infection appears to induce a similar response, albeit through the phosphorylation of different translation factors from those in mammals (109). However, this may be a common theme as DNA viruses are also known to induce, and to inhibit, the NIK1 kinase, which likewise phosphorylates translation-associated proteins, resulting in reduced cellular translation, which is detrimental to virus accumulation (reviewed in reference 94). These studies suggest that, like NLR-mediated responses, additional antiviral mechanisms may affect protein translation, and this area will be of great interest for further investigation.

## CONCLUDING REMARKS AND FUTURE DIRECTIONS

The 2025 Plant Virology Satellite Symposium highlighted the remarkable breadth and accelerating pace of discovery across the field of plant virology. From elucidating the mechanisms that govern transgenerational virus transmission to characterizing the complex viromes of economically important crops such as grapevine, the presentations underscored the increasingly sophisticated approaches being applied to understand virus–host–vector interactions. Emerging themes—such as the growing recognition of functional small viral proteins, advances in host resistance pathways, and the expanding diversity of viral vectors including mites—emphasize the dynamic and evolving nature of plant virus biology. The symposium showcased the translational potential of plant viruses, particularly through innovative nanotechnological applications that bridge plant and human health. These developments demonstrate how fundamental research on viral structure and replication can seed new strategies for disease management, crop protection, and even therapeutic design.

Importantly, the symposium grew from a community-driven effort to identify priority research areas and foster the inclusion of early-career researchers. This collaborative spirit reflects the broader mission of the American Society for Virology to cultivate a vibrant, interdisciplinary, and forward-looking scientific community. As global challenges such as food insecurity, climate change, and emerging plant diseases continue to intensify, the insights and technologies discussed at the 2025 meeting offer a strong foundation for advancing plant health and sustainable agriculture. The field of plant virology stands poised to contribute not only to scientific discovery but also to practical solutions essential for a healthier future.
